# Analyzing Land Use/Land Cover Changes Using Remote Sensing and GIS in Rize, North-East Turkey

**DOI:** 10.3390/s8106188

**Published:** 2008-10-01

**Authors:** Selçuk Reis

**Affiliations:** Aksaray University, Faculty of Engineering, Department of Geodesy and Photogrammetry, 68100, Aksaray, Turkey; E-mail: sreis@aksaray.edu.tr; sreis61@gmail.com; Phone:+90 382 2150953; Fax :+90 382 2150592

**Keywords:** Land use\land cover change, Landsat imagery, GIS, Rize

## Abstract

Mapping land use/land cover (LULC) changes at regional scales is essential for a wide range of applications, including landslide, erosion, land planning, global warming etc. LULC alterations (based especially on human activities), negatively effect the patterns of climate, the patterns of natural hazard and socio-economic dynamics in global and local scale. In this study, LULC changes are investigated by using of Remote Sensing and Geographic Information Systems (GIS) in Rize, North-East Turkey. For this purpose, firstly supervised classification technique is applied to Landsat images acquired in 1976 and 2000. Image Classification of six reflective bands of two Landsat images is carried out by using maximum likelihood method with the aid of ground truth data obtained from aerial images dated 1973 and 2002. The second part focused on land use land cover changes by using change detection comparison (pixel by pixel). In third part of the study, the land cover changes are analyzed according to the topographic structure (slope and altitude) by using GIS functions. The results indicate that severe land cover changes have occurred in agricultural (36.2%) (especially in tea gardens), urban (117%), pasture (-72.8%) and forestry (-12.8%) areas has been experienced in the region between 1976 and 2000. It was seen that the LULC changes were mostly occurred in coastal areas and in areas having low slope values.

## 1. Introduction

Land use/land cover (LULC) changes play a major role in the study of global change. Land use/land cover and human/natural modifications have largely resulted in deforestation, biodiversity loss, global warming and increase of natural disaster-flooding [14, 26, 7]. These environmental problems are often related to LULC changes. Therefore, available data on LULC changes can provide critical input to decision-making of environmental management and planning the future [8, 16].

The growing population and increasing socio-economic necessities creates a pressure on land use/land cover. This pressure results in unplanned and uncontrolled changes in LULC [18]. The LULC alterations are generally caused by mismanagement of agricultural, urban, range and forest lands which lead to severe environmental problems such as landslides, floods etc.

Remote sensing and Geographical Information Systems (GIS) are powerful tools to derive accurate and timely information on the spatial distribution of land use/land cover changes over large areas [4, 9, 17, 27] Past and present studies conducted by organizations and institutions around the world, mostly, has concentrated on the application of LULC changes. GIS provides a flexible environment for collecting, storing, displaying and analyzing digital data necessary for change detection [23, 6, 25]. Remote sensing imagery is the most important data resources of GIS. Satellite imagery is used for recognition of synoptic data of earth's surface [22]. Landsat Multispectral Scanner (MSS), Thematic Mapper (TM) and Enhanced Thematic Mapper Plus (ETM+) data have been broadly employed in studies towards the determination of land cover since 1972, the starting year of Landsat program, mainly in forest and agricultural areas [3]. The rich archive and spectral resolution of satellite images are the most important reasons for their use.

The aim of change detection process is to recognize LULC on digital images that change features of interest between two or more dates [15]. There are many techniques developed in literature using post classification comparison, conventional image differentiation, using image ratio, image regression, and manual on-screen digitization of change principal components analysis and multi date image classification [12]. A variety of studies have addressed that post-classification comparison was found to be the most accurate procedure and presented the advantage of indicating the nature of the changes [13, 24]. In this study, change detection comparison (pixel by pixel) technique was applied to the Land use\land cover maps derived from satellite imagery.

The aim of the study is to analyze LULC changes using satellite imagery and GIS in Rize Province (North East Turkey) neighboring to Black Sea. In order to achieve this objective, Landsat Multi-spectral Scanner (MSS) and Enhanced Thematic Mapper Plus (ETM+) data acquired on July 1976 and July 2000 were used. Maximum likelihood classification and change detection comparison strategy was employed to identify LULC changes.

## 2. Study Area

**Figure 1.**
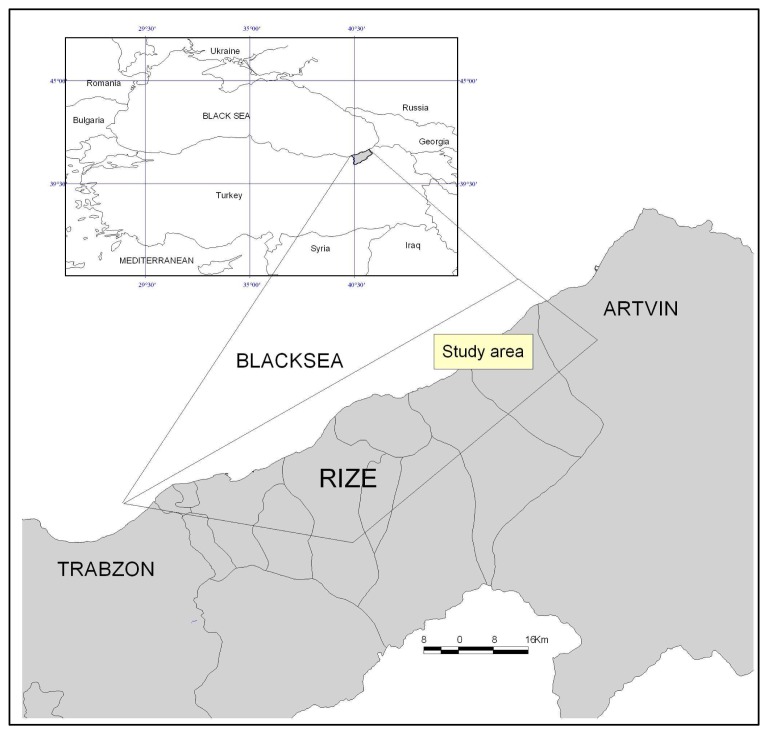
Location of the study area

The study area covers Rize which is located between 40°20′ and 41°20′ N latitudes and 40° 22′ and 41° 28′ E longitudes in the Northeastern Black Sea Region in Turkey. The area includes about a half of the Province of Rize (the whole coastal part of Rize and the area that reaches by 30 km of distance to the inner part, starting from the coast). The study area is characterized by a high steep and rough terrain with a mountainous area of 78% and an altitude changing from 0 to 2000 m. It is on the Northeastern Black Sea Region in Turkey covering an area of 2700 km^2^ (Figure 1). Rugged terrain is dominant in the province because of mountains that start to increase from coastal part to the Black Sea. The flat areas are very few except from the exits of narrow valley regions where the rivers reach to the sea [11].

**Figure 2.**
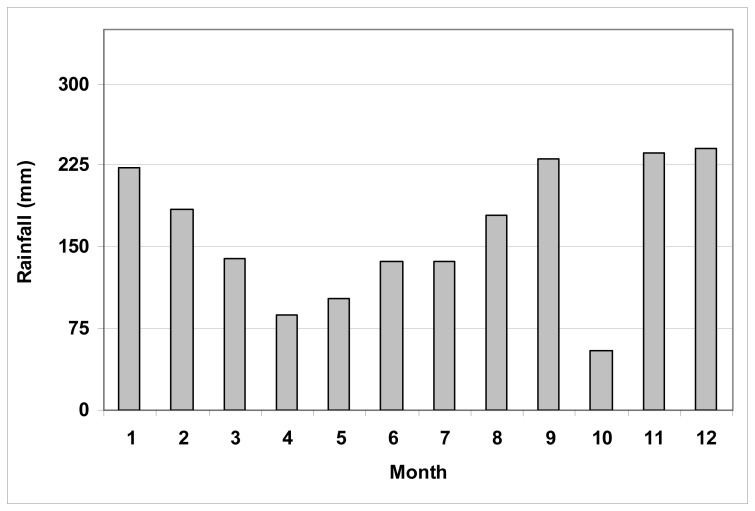
The mean annual rainfall of Rize region during the period of 1975 to 2000.

Between the years 1975 and 2000, the rainfall was observed between 1700-2700 mm and the average of annual rainfall was 2199.3 mm (average rainfall is 735 mm for Turkey). Figure 2 shows the mean annual rainfall in Rize between the years 1975 and 2000. The peak of rainfall amount is in October (301.3 mm), and the driest month is April (87.7mm). This region is the most rainfall area in Turkey. The coldest month is February (6,2 °C) and the hottest one is August(23 °C) in Rize. The mean of yearly temperatures, calculated based on a long period, is 14,1 °C [21].

Rize has a very rich hydrographic structure due to underground water resources. There are 25 rivers that are longer than 5 km in the boundaries of the Province of Rize. However only 16 of these rivers reach to the Black Sea and the rests are branch of one of those rivers.

Most of Rize province is covered by tea gardens and forest areas. The forest areas cover the most of the middle part of the whole province from east to west, while the tea gardens are mostly located in the coastal parts. Starting from 1917, tea plantation has been continuously increased up to present day [19].

Because of the geographical structure of the province, the residential areas are densely located throughout the coastal region and most of the populations live in that part of the province. Most of the population of the city lives in the residential areas which densely located throughout the coastal region.

On the other hand, since the suitable residential areas are inadequate, residences are scattered around in the inner parts of the province. While the population of Rize was 315.000 in 1970, it increased to 365.938 in 2000 [20]. Rize is one the geographical regions experiencing landslides and floods in Turkey. The essential reasons of landslides and floods occurred in the area are rain density, rough terrain, defrostration, improper reconstruction activities in the regions that are under the threat of natural disasters. Additionally, since the tea is one of the important agricultural products in Turkey and mainstay of people in the region, the tea agriculture has spread out and become prevalent in an unplanned and uncontrolled manner especially in forest areas by destroying forests.

## 3. Methods

### 3.1. Data

Landsat MSS and Landsat ETM+ (path 185, row 31) were used in this study. The Landsat ETM+ image (10 July 2000) was downloaded from USGS Earth Resources Observation Systems data centre and Landsat MSS image (09 July 1976) was provided by a commercial data provider. The dates of both images were chosen to be as closely as possible in the same vegetation season. 90 × 30 km of subset was used from these Landsat images. Landsat MSS has a 79 m and Landsat ETM+ 28.5 m spatial resolution. All visible and infrared bands (except the thermal infrared) were included in the analysis. Remote sensing image processing was performed using ERDAS Imagine 9.1.

Other materials used are aerial photos and standard topographic maps. The training sites and test sites maps, generated from aerial photo interpretation in 1973 and 2002, were digitized for the goal of creating a spatial database. Digital Elevation Model (DEM) was produced from the standard topographic maps with the scale of 1/25.000. DEM was created by using ArcGIS 9.2 GIS software. Slope and elevation maps were generated by using DEM. Pixel dimensions of this Slope Map are 50×50 m.

### 3.2. Remotely sensed imagery and pre-processing

Landsat 2000 image was geo-referenced (Universal Transverse Mercator-UTM, WGS84) to the map (topographic maps with the scale of 1/25.000 and produced by the General Directorate of Mapping), with an RMS error of less than 30 m by using nearest neighborhood resampling method. The Landsat 1976 image was then geo-referenced to the 2000 image (image to image registration), with an error of less than 80 m. The radiometric corrections and systematic errors were removed from the data set providers.

### 3.3. Image classification and accuracy assessment

In this study, totally, seven LULC classes were established as agriculture, bare soil, coniferous, deciduous, pasture, urban and water. Description of these land cover classes are presented in Table 1. Two dated Landsat images were compared supervised classification technique. In the supervised classification technique, two images with different dates are independently classified. Accurate classifications are imperative to insure precise change-detection results [10]. A Supervised classification method was carried out using training areas and test data for accuracy assessment. Maximum Likelihood Algorithm was employed to detect the land cover types in ERDAS Imagine9.1.

Accuracy assessment was critical for a map generated from any remote sensing data. Error matrix is in the most common way to present the accuracy of the classification results [8]. Overall accuracy, user's and producer's accuracies, and the Kappa statistic were then derived from the error matrices. The Kappa statistic incorporates the offdiagonal elements of the error matrices and represents agreement obtained after removing the proportion of agreement that could be expected to occur by chance [24].

## 4. Results and Discussion

### 4.1. Land use\land cover classification and accuracy

**Figure 3.**
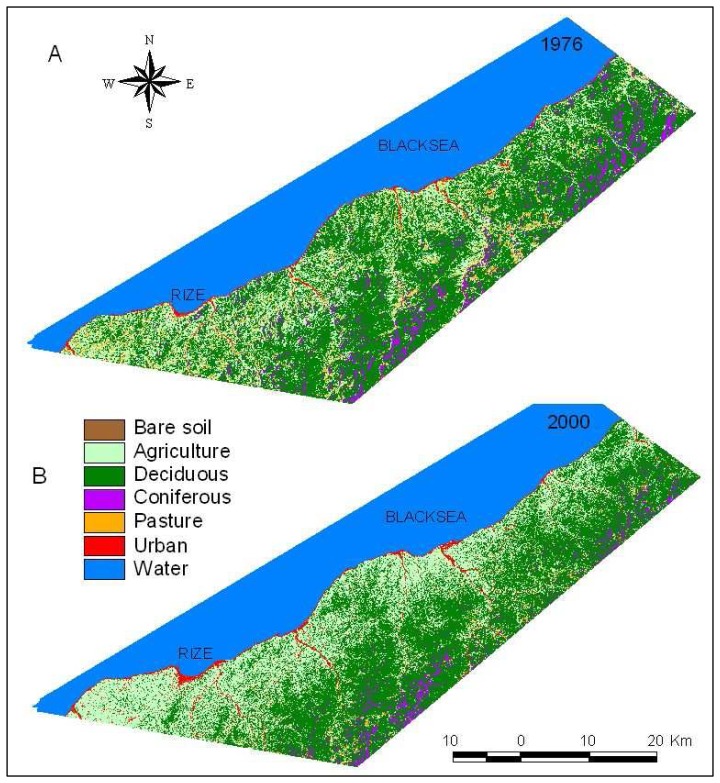
The classification images of land use\land cover in Rize (A: 1976, B:2000).

In this study, 104 polygons for Landsat ETM+ and 96 polygons for Landsat MSS 1976 were randomly selected to assess classification accuracy. Table 2 is the error matrix, along with the overall accuracy and the Khat coefficient. The overall accuracy of classification image dated 1976 was 84,4% and that of the image dated 2000 was 87,1% and the Kappa coefficient was 82.3% and 83,6%. In 1976 and 2000 classification, user's and producer's accuracies of individual classes were extremely high, ranging between 76% and 100%, and 75% and 100% respectively. The accuracies of classification were turned out to be better than expected. Classification operations became harder especially since there are only aerial images but no ground truth data that belong to 1973 and 2000. But having many tea gardens, located in coastal areas, made it possible to asses the classification results reliably. Since tea plant is short, tea gardens are sometimes located under deciduous with large leaves. Therefore, deciduous and agricultural classes (especially tea gardens) were mixture more than other classes. The classified images of land cover for 1976 and 2000 are shown in Figure 3.

### 4.2 Land use\land cover change in Rize

The LULC classification results are summarized for the years 1976 and 2000 in Table 3. From 1976 to 2000, agricultural areas, urban areas and bare soil increased 137030 ha (36.2%), 1859 (117.0%) and 2494 (174.9%) respectively. On the other hand deciduous, pasture, and coniferous decreased 7.029 ha (8.3%), 5.568 ha (72.8%) and 5.115 ha (50.2%) respectively. 60% of total tea production of Turkey was realized in study area [5]. Therefore, the changes in tea gardens in the course of time are meant by the changes lived in agricultural areas in 24 years. As seen in Table 3, while the areas well qualified for agriculture was 37859 ha in 1976, this number increased 36.2% and became 51562 ha in 2000. On the other hand there was a 12144 ha decrease in forest areas (deciduous and coniferous) in that those areas decreased from 94761 ha to 82617 ha. The increase in agricultural areas (13703 ha) and the decrease in forest areas (12144 ha) are approximately the same. These given data expressly state that the increase in agricultural areas mostly result in deforestation which means some forest areas were removed and converted to tea gardens in the region.

Agricultural lands were mostly located in the region with 0-500 m of altitude and a decrease occurred in agricultural area with the increase in altitude. Since the mountains start right after the sea coast, there are forest areas in the coastal region in study area. As these areas are mostly rugged, they can be used as neither residential nor agricultural areas. The forest areas are densely located in the regions with 1000 m of altitude in the study area, and the forest area is more than the other kinds of lands in places with high altitude.

**Figure 4.**
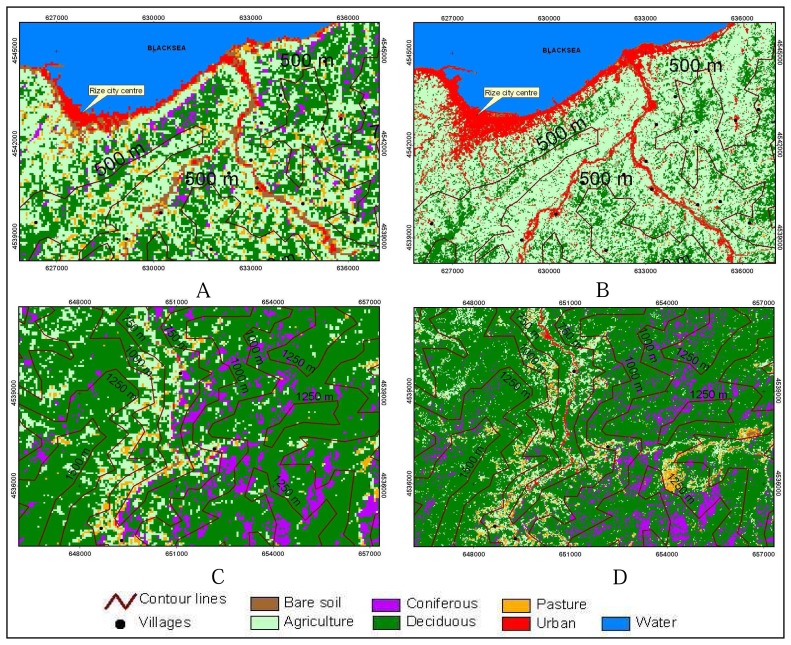
Example of Land use\land cover map with elevation, A) Coastal area of 1976, B) Coastal area of 2000, C) high mountainous area of 1976, D) high mountainous area of 2000.

The change detection matrix for the time period between 1976 and 2000 was produced using pixel by pixel method (Table 4). The lands converted in to agriculture area are covered by mostly deciduous (22680 ha), pasture (3987 ha) and coniferous (1144 ha). As seen in change matrix the lands converted to agriculture area are mostly the lands covered with deciduous plants. These lands were converted in to tea fields in especially densely populated coastal regions (Figure 3 and 4). On the other hand the lands converted to the deciduous class are mostly agriculture, coniferous and pasture land classes. This conversion especially occurred in tea fields in rural areas because of the migration to the cities. This issue is explained is section 4.3 in more details. It is clearly seen in the matrix that coniferous and deciduous classes are mixed. The most important reason of this is that coniferous and deciduous classes are one within the other. The lands converted to the bare soil class are mostly agriculture, deciduous and pasture classes. As seen in the matrix the bare soil determined in both 1976 and 2000 are quite less. This may stem from the changes of bare soil location in the course of time. These lands usually consist of filled land in the coastal areas, and sand and gravel areas in the bank of streams. Since the coasts were expanded by filling materials, the bare soil in these regions hit in different places in 1976 and 2000. Similarly, since the lands determined to be bare soil in the bank of streams in 1976 were converted to residential areas in 2000, the size of the overlapping areas were calculated to be quite less. Moreover, except from tea fields, the agricultural areas in the form of arable fields formed in the areas close to the residential regions were converted in to bare soil in 2000. 5568 ha of pasture areas were disappeared because of the pressure of agriculture and deciduous class of lands. There are conversions to urban class from all kinds of classes of lands, but the conversion from coniferous was relatively less. A long with the increase in population, the area of Rize city center was enlarged by the conversion of neighboring agriculture areas in to residential areas. Similarly, because of the structuring pressure on river bank regions and coastal areas, bare soil in those areas was converted in to residential areas. The water areas converted in to residential areas were originated from filling the coastal areas and opening those places for housing.

The areas that changed in the course of time are mostly located in the coastal areas. As a matter of fact the changes in lands from forest to agricultural area are experienced in these regions most. This increase is denser in the regions extending to inner parts about 5 km from the coast. In the surrounding area of city center of Rize and its west parts, the increase in agricultural areas was seen more. It is because of the fact that tea plantation and production were fostered by government between the years 1960 and 1990. Since the region's economy was already relied on tea production, this support resulted in a decrease in forest areas, especially in the surrounding sections of the residential areas in an uncontrolled manner. Urban areas also increased with a ratio of 117% in the study areas along with the increase in population. This increase mostly occurred in the coastal parts and within the centers of districts (Figure 4). On the other hand, some changes occurred in pasture areas, located in the region with 1500-2000 m of altitude, in the study region in the course of time. In this context, approximately 5.568 ha of decrease occurred in pasture areas and 2.494 ha of increase occurred in bare soil. A little bit pasture area was naturally turned in to forest as some trees were planted in the pasture.

### 4.3. Analyzing LULC changes according to topography

**Figure 5.**
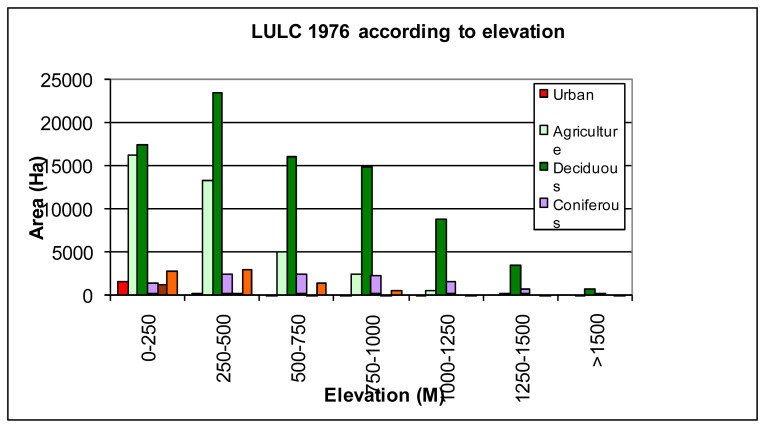
Land use\land cover values that belong to 1976 according to altitude.

The relationship between land use\land cover and topography, in the time period of 1976-2000, was analyzed by using Digital Elevation Model (DEM). Before all else, how land use\land cover was changed in the course of time was determined according to elevation. The results of analysis of land cover in 1976, performed according to altitude, are given in Figure 5. According to Figure 5, residential areas are mostly located in regions with 0-250 m of altitudes. It is because of the fact that the region is quite rugged, and as a result of this rough structure, only small flat areas, located in the coastal sections, and valley channels can be used as residential areas. Transportation is one of the important problems for the region because of rugged topographic structure [2]. Therefore, Rize city center and districts are located in the coastline. During the classification of Landsat MSS image data, it was impossible to detect the small residential places due to low spatial resolution of MSS image in the region where scattered residential properties are dominant.

**Figure 6.**
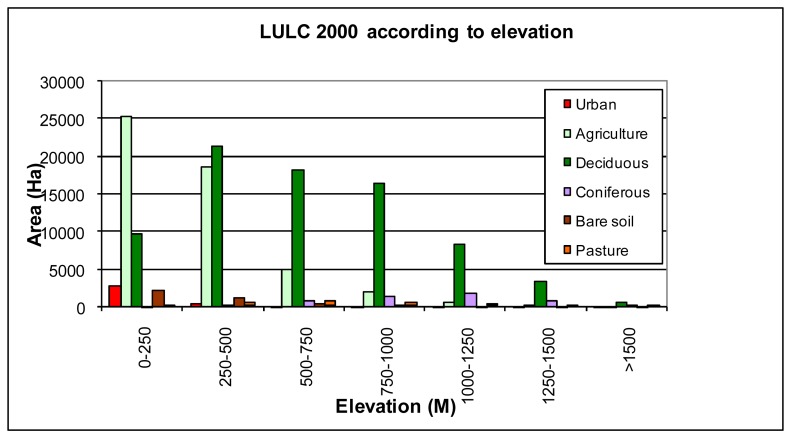
Land use\land cover values that belong to 2000 according to altitude.

The LULC for 2000, which are formed according to the altitude, are seen in Figure 6. Agricultural areas were densely located in regions with 0-500 m of altitude as it was in 1976. The values show that agricultural areas are decreased with the increasing altitude (Figure 4). It is because economic and social living conditions are harder in small residential places located in regions with high altitude. Areas that can be used for agricultural activities are also very limited. Therefore, the population of rural villages located in regions with high altitude is very low. Along with the industrialization process, starting from 1970s, an important migration movement took place from rural regions to the metropolitan cities and this migration process has not ended yet [1]. Consequently some areas, used for agricultural activities in patches in 1976, naturally turned in to forest areas when it came to the year of 2000. Likewise, forest areas are denser in rural sections located in the regions with 0-1250 m of altitude. Bare soil class is decreased with increasing altitude.

When the changes in residential areas are compared according to altitudes, distinctive residential properties can be found out. The study shows that residential areas were located near the coast (0-250 m) in 2000 and 1976. Besides, residential areas were mostly located in the regions with 0-250 m of altitude in 24 years time period. The agricultural areas were also increased in coastal regions (0-500 m). However, forest areas were decreased in the region with 0-500 m of altitude, but they were increased in the regions with altitude higher than 500 m (Figure 4). While the bare soil areas were decreased in coastal regions, they were increased in higher rural parts. Pastures on the other hand were decreased in all regions with different altitudes.

**Figure 7.**
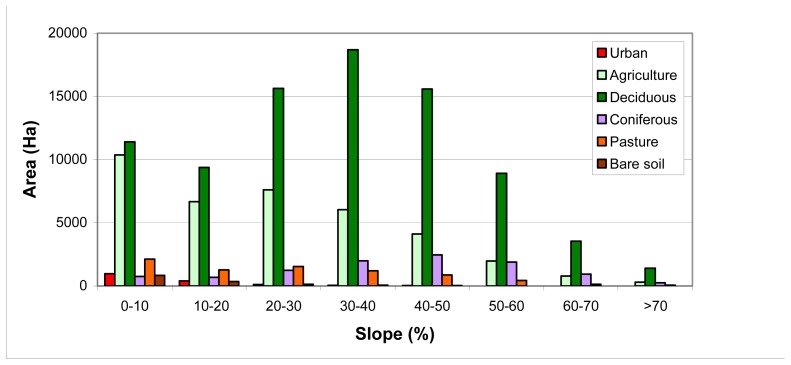
Land use\land cover and Slope in 1976

**Figure 8.**
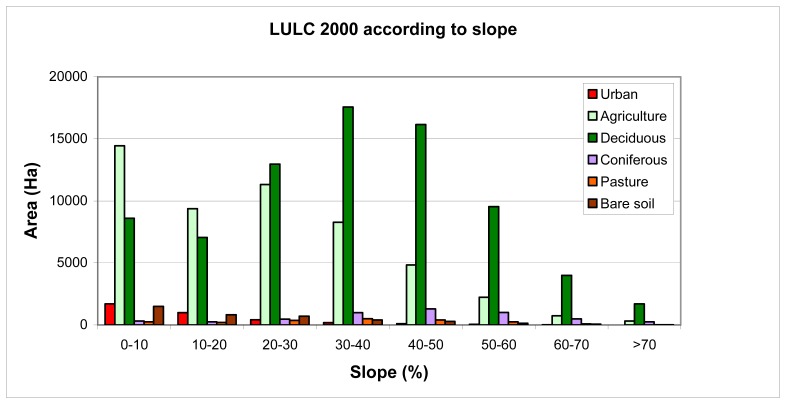
Land use\land cover and Slope in 2000

The study area is a quite sloping region. Therefore, land cover changes are investigated according to slope in Figure 7 and 8. Densely populated residential areas fall in to the region with 0-20% slope value in 2000 and 1976. But in 2000, the residential areas were fairly increased in regions with 20-50% slope value. In 24 years time period, while the residential areas were increased 200% in regions with 0-20% slope value, the rate of increase were 330% in regions with 20-50% slope value. Forest areas (deciduous and coniferous) are having lower slope value were most densely located in regions with 20-60% slope values. It is seen that forests that fall in to 0-30% slope value interval were decreased in 2000. Despite this, Figure 8 shows that forest areas were increased in the regions with a slope value higher than 40% when compared to 1976. It is because of the fact that, since the areas with lower slope values is extremely limited in the region; these areas were mostly used for agricultural activities or for residential purposes. Pasture areas were generally decreased in all the regions with different slope values.

## 5. Conclusion

This paper aims investigating land use/land cover changes occurred in Rize between 1976 and 2000 using remote sensing and GIS. The LULC changes were analyzed according to both slope and altitude. The main change observed for the time period of 1976-2000 was that the area of agriculture (mostly green tea) was increased approximately 13700 ha, and forest area was decreased approximately 12100 ha. 60% of total tea production in Turkey was performed in the study area. Since the tea agriculture was fostered by government between 1960 and 1990, while the agricultural areas were increased in lands near coastal sections in an uncontrolled form, forest areas were decreased in the same rate. Especially coastal sections and valley channels are the mostly changed regions. In the related and low sloping regions (0-20%), forest areas were decreased contrary to increasing agricultural area. The regions having high altitudes were exposed to totally reverse changes. In regions having altitude higher than 750m, contrary to decreasing agricultural areas, forest areas were increased. The most important reason for this is that the migration from rural areas to urban areas. Since there were very limited and uneconomic agricultural areas in rural areas located in regions with high altitudes, and some tea gardens were abandoned, those unexploited areas were naturally turned in to forest areas. Moreover, the climate pattern of the region is very suitable for forestation.

Another subject is some difficulties faced in determining LULC using remotely sensed data in the study area. The mountainous and sloping topographic structure of the region and complex vegetation of the area and negative climate conditions are the essential reasons for those difficulties. For this reason, it was quite hard to find usable (not cloudy) satellite images. There were also some other problems that had stemmed from using different sensor technologies (spatial resolution and spectral resolution) in comparing Landsat MSS and ETM data, and in determination of land cover. These problems were tried to be eliminated by independently applying supervised classification change detection technique to both images.

## Figures and Tables

**Table 1.** Land cover classification scheme

Land Cover Classes	Description
Pasture	Areas consisting of arid lands with short vegetations
Bare soil	Areas with no vegetation cover, stock quarry, stony areas, uncultivated agricultural lands
Water	Sea, river, lake
Urban	Residential, commercial, industrial, transportation and facilities
Agriculture	Almost all are green tea gardens, small size corn and fruit gardens that are generally located in tea gardens
Coniferous	Coniferous forest, trees that don&apost patch off (spruce scotch pine etc)
Deciduous	Deciduous forests, mixed forests with higher density of trees (alnus tree, linden tree, chestnut tree etc.)

**Table 2.** Accuracy assessment for supervised classification of Landsat MSS 1976 and Landsat ETM+ 2000

Accuracy assessment of the Landsat MSS 1976									
**LULC**	**A**	**D**	**C**	**BS**	**P**	**U**	**W**	**Total**	**PA (%)**
**A**	429	53	0	0	11	0	0	493	87,0
**D**	46	627	45	0	6	0	0	724	86,6
**C**	0	95	395	0	0	0	0	490	80,6
**BS**	9	0	0	143	22	12	0	186	76,9
**P**	36	9	0	8	210	0	0	263	79,8
**U**	2	1	0	21	3	254	0	281	90,4
**W**	0	0	0	0	0	0	152	152	100,0
**Totals**	522	785	440	172	252	266	152	2589	
**CA (%)**	82,2	79,9	89,8	83,1	83,3	95,5	100,0	85,4	
**Overall accuracy**=84,4% ,**Kappa coefficient**=82,3%									
Accuracy assessment of the Landsat ETM+ 2000									
**LULC**	**A**	**D**	**C**	**BS**	**P**	**U**	**W**	**Total**	**PA (%)**
**A**	685	100	0	14	3	1	0	803	85,3
**D**	92	969	26	3	0	1	0	1091	88,8
**C**	2	107	331	0	0	0	0	440	75,2
**BS**	20	2	0	251	12	12	0	297	84,5
**P**	27	7	0	9	246	0	0	289	85,1
**U**	4	0	0	38	4	750	0	796	94,2
**W**	0	0	0	0	0	0	52	52	100,0
**Totals**	830	1185	357	315	265	764	52	3768	
**CA (%)**	82,5	81,8	92,7	79,7	92,7	98,2	100,0	87,1	
**Overall accuracy**=87,1%, **Kappa coefficient**=%83,6									

Note: A=agriculture, D=deciduous, C=coniferous, BS=bare soil, P=pasture, U=urban, W=water

**Table 3.** Summary of Landsat classification area statistics for 1976 and 2000

Land cover class	1976		2000		Relative Change	

	ha	%	ha	%	ha	%
Agriculture	37859	17.4	51562	23.7	13703	6.3
Deciduous	84577	38.8	77548	35.6	-7029	-3.2
Coniferous	10184	4.7	5069	2.3	-5115	-2.4
Bare soil	1426	0.7	3920	1.8	2494	1.1
Pasture	7645	3.5	2077	1.0	-5568	-2.5
Urban	1589	0.7	3448	1.6	1859	0.9
Water	74689	34.3	74345	34.1	-344	-0.2

**Table 4.** Change matrix of land use land cover (ha) in 1976 to 2000

Year	**1976**									
	**Land cover class**	**A**	**D**	**C**	**BS**	**P**	**U**	**W**	**Total**	**Change rate (%)**
2000	**A**	**22896**	22680	1144	402	3987	452	1	51562	36.2
	**D**	11700	**56997**	6604	92	2065	51	39	77548	-8.3
	**C**	102	2724	**2204**	3	33	3	0	5069	-50.2
	**BS**	1594	1001	125	**135**	828	141	96	3920	174.9
	**P**	682	867	85	10	**422**	10	1	2077	-72.8
	**U**	880	300	22	579	310	**923**	434	3448	117.0
	**W**	5	8	0	205	0	9	**74118**	74345	-0.5
	**Total**	37859	84577	10184	1426	7645	1589	74689	217969	

Note: A=agriculture, D=deciduous, C=coniferous, BS=bare soil, P=pasture, U=urban, W=water
